# Protecting contacts against privacy leaks in smartphones

**DOI:** 10.1371/journal.pone.0191502

**Published:** 2018-07-11

**Authors:** Youngrok Cha, Wooguil Pak

**Affiliations:** Department of Computer Engineering, Keimyung University, Daegu, Republic of Korea; University of Connecticut, UNITED STATES

## Abstract

Due to recent developments in technologies associated with the Internet of Things (IoT), a large number of people now regularly use smart devices, such as smartwatches and smartphones. However, these devices are prone to data leaks because of security vulnerabilities. In particular, Android devices use permission-based security, which allows users to directly approve permissions requested by an app when installing it. As a result, many malicious apps can obtain and leak private user data by requesting more permissions than are needed. However, it is difficult to identify malicious apps based solely on the requested permissions. A system is hence needed to accurately identify malicious apps and protect private data from them. In this paper, we propose a system for hiding data related to a user’s contacts or providing virtual data according to preconfigured policies when an Android app requests access to them. By hiding data related to the contacts, the proposed system can protect them from malicious apps. By using virtual data, it can even detect malicious apps that leak private data. The system requires less storage and provides faster access to user contacts than prevalent solutions to similar problems.

## Introduction

Android is the most popular operating system for smartphones worldwide with a market share of approximately 86.1% [1]. Since it is open source, anyone can use and modify the source code. This feature enables Android to be used anywhere and on any device. However, open-source software can be more vulnerable to malicious users and apps than closed proprietary platforms, and Android has accordingly suffered from various security threats in recent years, such as private data leaks [2–17].

Many malicious apps nowadays seek to access and use private data on smartphones without this being noticed by users. Leaked private data are used mostly for e-mail spam and voice phishing. E-mail spam has significantly increased in volume over the years, and is a serious problem as it is costly for users, companies, and even governments.

Some cellphone carriers have lately begun providing a service called ‘intelligent spam filtering’ to customers to help them avoid spam [18–20]. However, it is well known that its effect is limited because it relies on pattern matching based on a pre-built database containing information about known spammers. Hence, it cannot effectively handle unknown attacks. The only viable solution is to prevent the leaking of private data in the first place.

In this paper, we propose a new approach to protect private user data from malicious apps. It extends the original contacts of a user on an Android device to hide private data from untrusted apps or share virtual fake data with them instead of real data. By doing so, private data leaks can be avoided. Our approach also provides a solution for safely running untrusted apps without having to worry about leaks of private data even when apps require access to the data. It can also detect private data leaks and accurately identify the guilty app. Although our approach is similar to virtualization-based solutions, it has many advantages that are not achievable by these methods, such as small storage requirement and fast access.

The remainder of this paper is organized as follows: In Section 2, we briefly review related work on Android security. We describe the overall structure of the proposed system and each core component in detail in Section 3, and report tests on our approach through intensive experiments in Section 4. We offer our conclusions in Section 5.

## Related work

Research on leakage of private data can be divided into studies that have focused on detecting malicious apps and those that have considered protecting internal private data by supplementing the system. The detection of malicious apps can be further divided into static approaches, which analyze the app package itself, and dynamic approaches, which analyze the execution behavior of the app.

### Static analysis

Static analysis-based approaches determine whether apps are malicious or benign, and consider the possibility of leakage of private data by analyzing the package files of apps without running them [21–36].

Some well-known static approaches are those based on ‘signature’ and ‘code analysis.’ Signature-based approaches [32] detect malware by using pattern matching with a signature database built beforehand by analyzing known malicious apps. Code analysis-based approaches determine the possibility of data leaks by decompiling and analyzing the ‘.dex’ files of the suspected app. Another approach uses the ‘AndroidManifest.xml’ file to obtain privileged information to determine whether a given app is malicious.

Yet another static approach based on a control flow graph (CFG) detects user privacy leaks through inter-component communication (ICC) [33–35]. A machine learning-based approach was recently proposed [36] that uses Extra-Trees, a machine learning algorithm to detect malicious apps regardless of code obfuscation.

There is a new approaches to combine static analysis with recommendation algorithm. It recommends selected apps according to app risk score calculating method (ARSM) which take account of statically analysis results for apps’ permissions and users’ interests [37].

Since static analysis can determine whether apps are malicious as they are being installed, it can prevent damage from malicious ones. However, it has critical limitations as it cannot accurately detect malicious apps if they exploit permissions by collaborating with pre-installed apps [38]. Moreover, code obfuscation and dynamic code execution deteriorate the performance of static analysis-based approaches. For these reasons, techniques based on static analysis struggle to detect malicious apps.

### Dynamic analysis

In contrast with static analysis, dynamic analysis involves running an app to check for private data leakage. To analyze the dynamic behavior of the app, it collects various logs while running the app, trails information flow between content providers, and monitors the system calls made by the app [39–60].

Dynamic analysis takes a long time because it needs to wait for the malware to misbehave. It sometimes adopts a virtual environment to reduce the time needed for analysis and yield more accurate results. However, if the app is programmed to leak private data under specific conditions, dynamic analysis has difficulty to detect malware.

TaintDroid is a well-known anti-malware system based on the dynamic approach [59]. It detects privacy leaks through ICC and informs the user of the system in real time. A dynamic approach based on machine learning has recently been proposed. For example, there was a research exploiting two machine learning algorithms, a linear classifier and a support vector machine, to achieve high classification accuracy in identifying malicious apps [60].

In the original Android, it is nearly impossible to accurately trace the flow of private data. Therefore, Android should be extended to track such access flow by attaching labels to it. However, this requires modifying the Dalvik virtual machine or the kernel, which degrades the performance of the system due to labeling overhead.

### Enhancing system security

This approach strengthens system security to render it immune to malicious apps. SEAndroid is a well-known derivation of SELinux to Android [61]. It adopts mandatory access control (MAC) based on a predefined policy. In SEAndroid, each process and object belong to each specific domain, and the policy defines the domains that can access particular objects [62]. It is effective in privilege escalation of vulnerabilities in the existing Android security model. However, the main obstacle to using SEAndroid is that no efficient solution has been developed to date to configure its complicated policy.

To improve system security, a 'purpose involved access control framework' (PACF) was proposed [63]. Previous access control frameworks focused on who performs which action on which data. Thus, privacy policies are defined by user, action, and data. However, PACF focuses on user's purpose itself to eliminate the limitation of previous systems. Some researches have been proposed to protect privacy data using PACF [64–66]. Complicate policies for privacy are defined in terms of ‘purpose’. Administrator can preserve private data through PACF to restrict accesses.

Another approach to extending the Android system involves allowing users to formulate policies for the access control of each app [67]. MockDroid is the most well-known system exemplifying this approach. It can protect private user data from all apps installed on smartphones. Permissions for accessing private data can be configured as normal or mocked. Normal permissions allow an app to access to private data according to the Android security mechanism. If a permission to access private data is configured as mocked, an app obtains empty data due to missing data or hardware limitations whenever it requests data. Since it hides all private data, MockDroid provides tight security to users. However, some apps fail to work properly with empty data.

A recent study proposed an approach to control the private data available to apps using virtualization technology [68]. An app running on each virtualization instance can only access the resources for that instance. Thus, it is among the most effective solutions to prevent the leakage of private data [69–75].

Since mobile devices have limited resources, container-based virtualization is used instead of system virtualization because of its low overhead. Samsung KNOX and VMWare AirWatch are well-known commercial products in this vein [74, 75]. However, virtualization encounters the problem of resource redundancy. For example, containers A and B should have two independent contacts. Therefore, it consumes more storage and memory. It causes another limitation not to support multiple containers. Most virtualization solutions, including KNOX support, only one or two containers at most. Thus, they are limited at managing private data for various apps.

## Proposed approach

Although various techniques such as static analysis, dynamic analysis, and enhancing system security have been developed, the fundamental solution against malicious apps does not exist. Since malicious apps have been evolved and many variants have created to avoid security solution, it is impossible to defend private data against such variants in time by updating existing security solution. Thus, we propose a new approach which deceives malicious apps by providing fake privacy data or hide real ones. In this case, malicious apps can leak only fake information, so we can keep privacy safe. If we monitor fake information, it is possible to detect leaking easily. Moreover, if we can elaborately design the information, it can help users to find malicious apps.

The proposed system belongs to the system security enhancement approach. However, it is designed to increase the security of the system without incurring redundant resources for existing virtualizations.

### Features of the proposed approach

In the proposed system, a user classifies each app into predefined categories according to its reliability. For each category, the user configures a policy to determine the private data that are made accessible to apps in it. The system provides fine granularity to control private data and, therefore, can prevent or minimize the damage caused by malicious apps.

The proposed approach is similar to SEAndroid in that it provides policy-based access control, but it is also similar to container-based virtualization in that the private data exposed to apps are independent for each category to which the apps belong. The proposed system can be configured to hide all or part of private data, or to expose virtual data instead of real data when the app is unreliable.

#### Private data hiding

The proposed system uses virtual data, and is very similar to the safe number service used in online shopping [76]. For example, an e-mail sent to a virtual e-mail address is relayed to the user’s actual e-mail address by an external e-mail proxy server. Virtual phone numbers are also used to replace real ones, and can be discarded when no longer needed.

Any function requiring private data can be seamlessly served such that the user can use even malicious apps without worrying about leaking private data.

The e-mail proxy server can add a warning message to the original e-mail to highlight spam e-mails. The virtual phone number server can also deliver a warning message before call setup to let the receiver know that the phone number has been leaked and may be used for such crimes as voice phishing.

#### Leak detection and path tracking

In addition to protecting private data from security threats, the proposed approach detects the leakage of such data and tracks how they are leaked. For example, if a user enables virtual data for a contact, each app belonging to different category gets different virtual private data. It is assumed that specific virtual e-mail information is leaked by a malicious app and used for e-mail spam. The user notices that private data has been leaked when the e-mail proxy server receives spam addressed to the virtual e-mail address. The user can also find the app category to which the malicious app belongs from the virtual e-mail address of the spam e-mail. App category is helpful to detect malicious app.

### Overall structure

**Fig 1:**
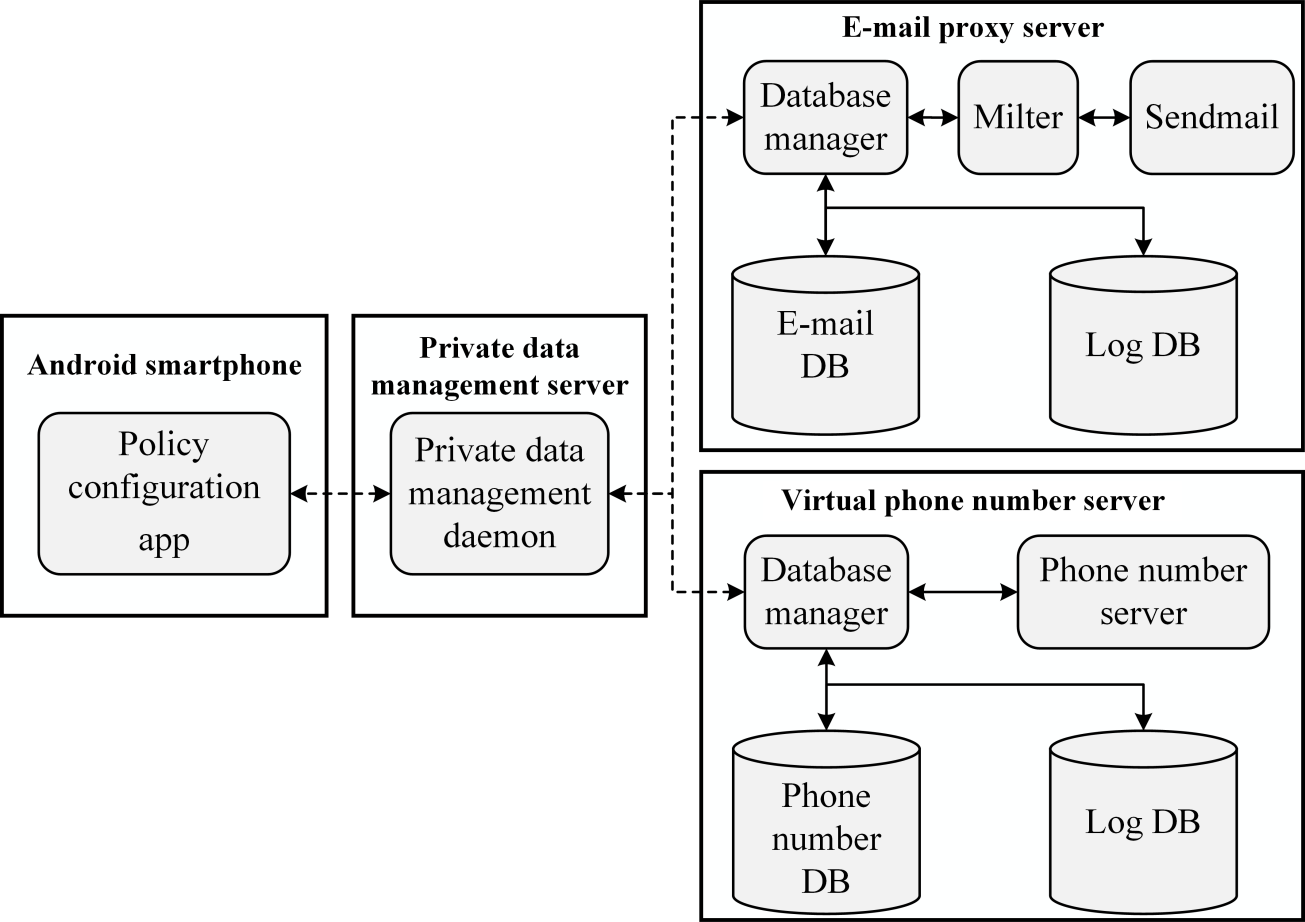
Block diagram of our proposed system.

The overall system structure is shown in Fig 1. Android smartphones only connect to the private data management server when the user configures the policy using the policy configuration app on a smartphone.

If the management server detects private data leakage or related events, it sends a message to the smartphone to notify the user. We now explain each core component of the proposed system.

### Policy configuration app

**Fig 2:**
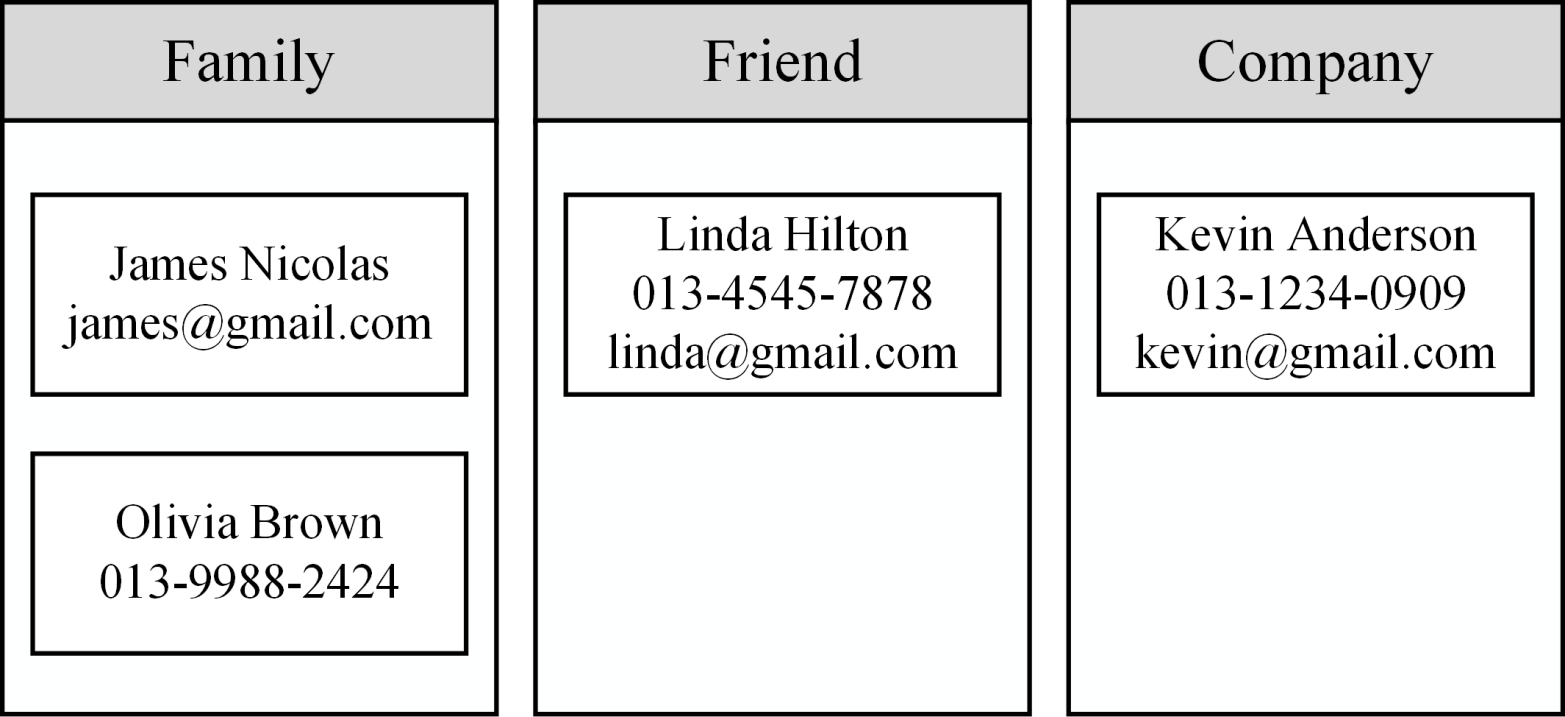
An example of the categories of contacts. Information pertaining to four contacts is in three categories.

Data in the contacts’ list, the address book of Android, are classified into several categories, called the contacts’ category, according to the required security level. The installed apps are also grouped into categories, i.e., app categories, according to the level of trust in them. Fig 2 shows an example where the user has classified contacts’ data into ‘family,’ ‘friend,’ and ‘company’ using a policy configuration app.

**Fig 3:**
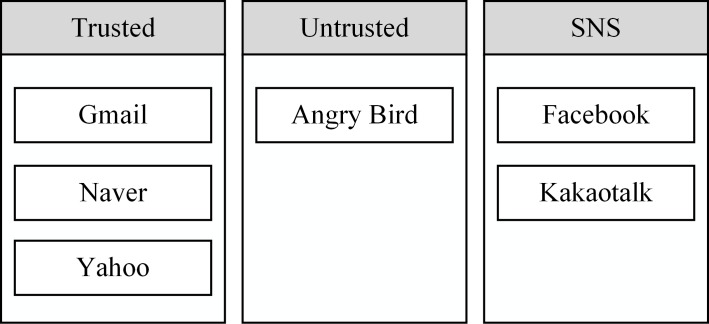
An example of app categories for the installed apps. Six apps are classified here into three categories.

Fig 3 also shows that all installed apps are divided into three groups of ‘trusted,’ ‘untrusted,’ and ‘social network service’ (SNS) apps. We use three categories in Figs 2 and 3 as examples, but there is no restriction on the number of contacts and app categories.

If we use more categories, it is easier to precisely detect malicious apps leaking information, but management and storage costs increase. Therefore, users should select the numbers of categories of app and contacts according to their needs.

Once the contacts and apps have been categorized, the user sets up a policy to determine how data relating to contacts belonging to each contact category are shown to apps in each app category. Therefore, a policy is assigned for each pair of contact and app category, denoted by [*C*,*A*] where *C* and *A* represent the names of the contact and the app categories, respectively.

- No-protection
  When the policy is set to ‘No-protection’ for [*C*,*A*], the contacts’ data belonging to *C* are directly shown to any app belonging to *A*. Therefore, it is used only for trusted apps or unimportant contacts’ data.
- Hiding
  When the policy is set to ‘hiding’ for [*C*,*A*], the contacts’ data belonging to *C* are invisible to any app belonging to *A*. It is used to prevent apps in *A* from leaking data in *C*.
- Virtualization
  When the policy is set to ‘virtualization’ for [*C*,*A*], the contacts’ data belonging to *C* are invisible to the apps belonging to *A*. Instead, virtual contacts’ data are exposed to the apps.

The virtual data are synthesized by the private data management server shown in Fig 1, and are uniquely used for [*C*,*A*]. Thus, if ‘virtualization’ is the policy for both [*C*,*A*_1_] and [*C*,*A*_2_], app *X* in *A*_1_ and app *Y* in *A*_2_ receive different virtual data corresponding to the data in *C*.

**Fig 4:**
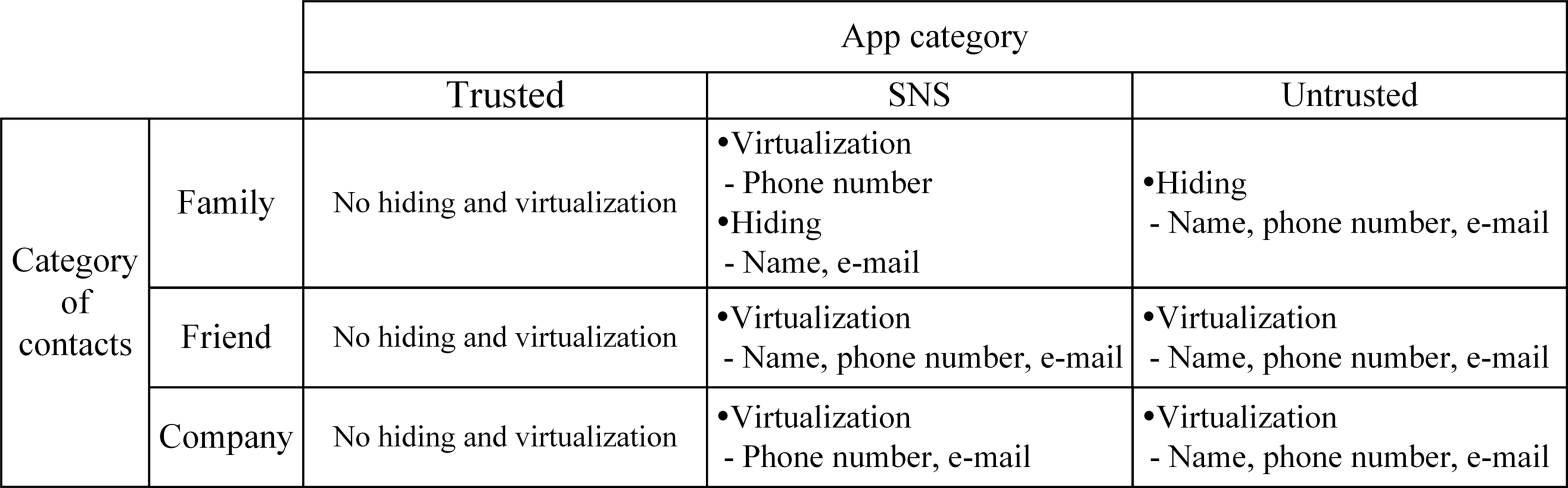
Contacts in the untrusted apps’ category.

Fig 4 shows an example of policy configuration where the apps only in the trusted apps’ category access the original contacts’ data. For apps belonging to other categories, part of the contact data are replaced by virtual data or hidden according to the policy.

### Structure of contacts’ database to support policy

In an Android system, an app receives the same contacts’ data when it accesses the contact list. However, in the proposed system, each app receives different data as determined by the policy. To this end, it needs an independent and separate contacts’ data for each app category. However, the total size of the contacts’ data increases proportional to the number of app categories, hence incurring significant storage cost.

In another approach, an integrated contacts’ database can be used to support the above feature. The integrated database can be built by adding data for the app categories for the contacts. It can help save storage but increases access time, as the system needs to dynamically generate data for apps according to the relevant policies.

To solving the above issue, we propose an approach that uses a ‘view’ for each app category. View is a virtual table and represents a subset of data in the ‘table’, but it takes up less storage but achieves almost the same access time as a table in SQLite in Android [77]. Hence, it can simultaneously solve the issues of storage space and access time. We will describe the structure of the contacts’ database in detail.

(1) A single database

One of the simplest ways to implement the proposed approach is by using separate contacts’ databases for each category. However, this consumes more storage and increases access time. To solve this problem, the original and virtual data are stored together in an integrated database, which helps reduce the requisite storage size and access time. However, it requires a more elaborate database structure.

(2) Prebuilt contacts’ view

When an app requests access to contacts, the proposed system not only searches the original data, but also virtual data if required according to the policy. It then merges them into one large view and reveals them to the requesting app. However, this causes a delay in processing the procedures when the app requests access to data. To avoid this, we extend the Android middleware to build ‘view’ in advance. The extended Android creates a ‘view’ when an app belonging to a specific category is launched for the first time. The Android system needs to maintain the view until all apps belonging to the same category are closed. Once the view has been created, the app accesses it instead of the contacts’ database table. The view is almost identical to the table in terms of functionality but consumes very little memory.

If a user creates a large number of app categories, the total memory required becomes a very important factor for scalability. Because of the small size of the ‘view,’ the proposed system can support a large number of app categories.

We consider an example to clarify the above. A user configures a policy for an untrusted app as shown in Fig 4. When the user first launches any app from the untrusted app category, e.g., the game Angry Bird, the proposed system creates a ‘view’ of contacts for untrusted app categories.

If Angry Bird tries to access the contacts, the Android system redirects access to a private ‘view’ instead of the existing contacts’ database table. By doing so, no app in the category can access the original contacts’ table and, therefore, private data are protected.

(3) Structure of fast access-oriented table

**Fig 5:**
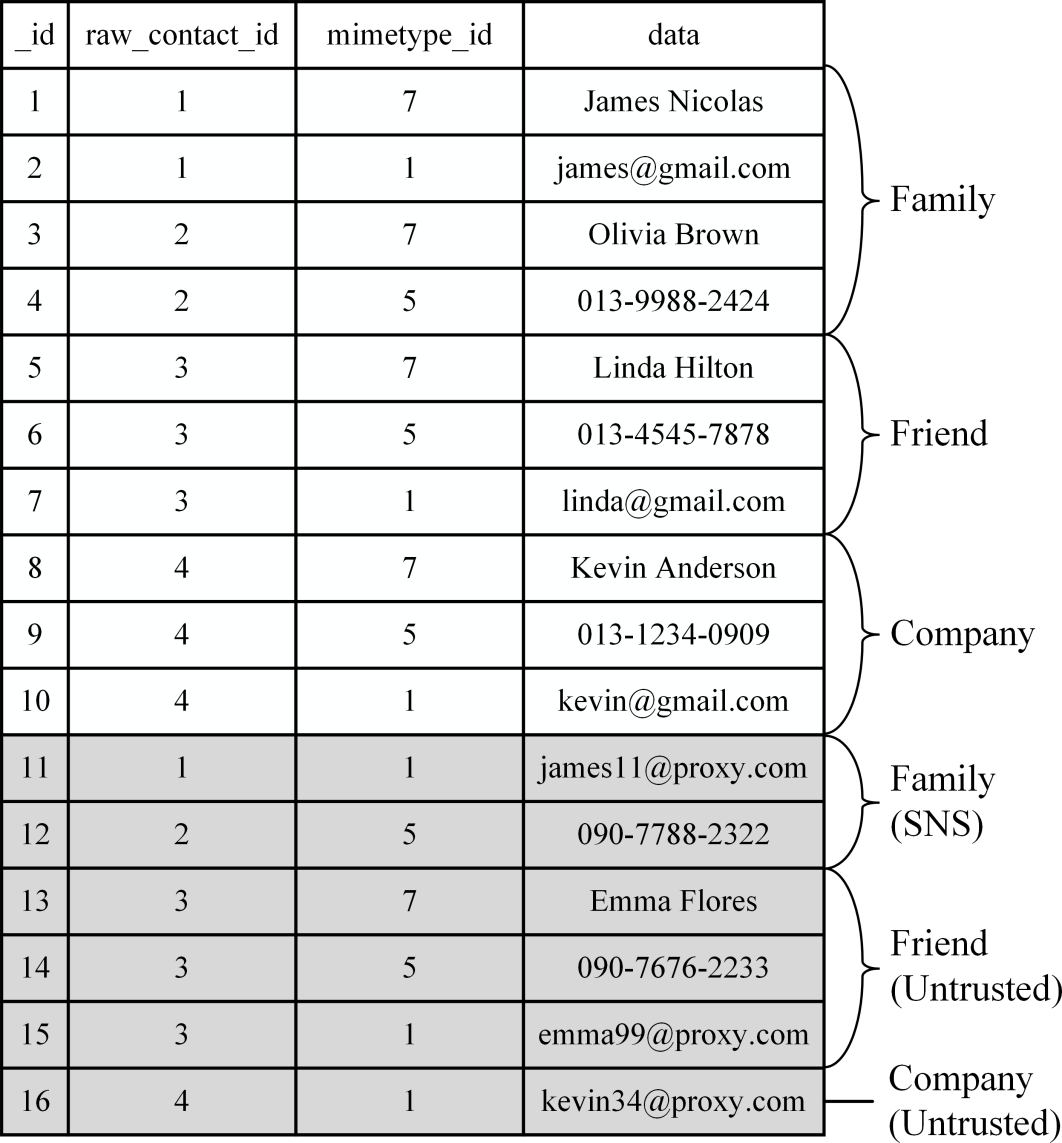
An example of the proposed database structure. The gray-colored records denote virtual data.

In the above example, when a user runs an app, Android creates a ‘view’ if needed. For this, it selects the required records in a contacts’ database for original and virtual data according to the relevant policy as they are stored in the same database. Assuming that each record is scattered all over the database, it takes a long time to create a view. In the proposed system, we organize the original and virtual data for fast access. Contacts’ data in the database configured as original or virtual data and belonging to the same contacts’ category are consecutively located when they are sorted by a primary key ‘_id’ as shown in Fig 5.

**Fig 6:**
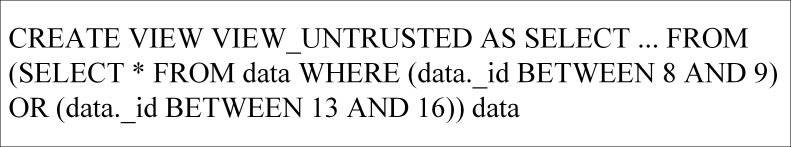
SQL query to create a “view” for an app in the untrusted app category.

**Fig 7:**
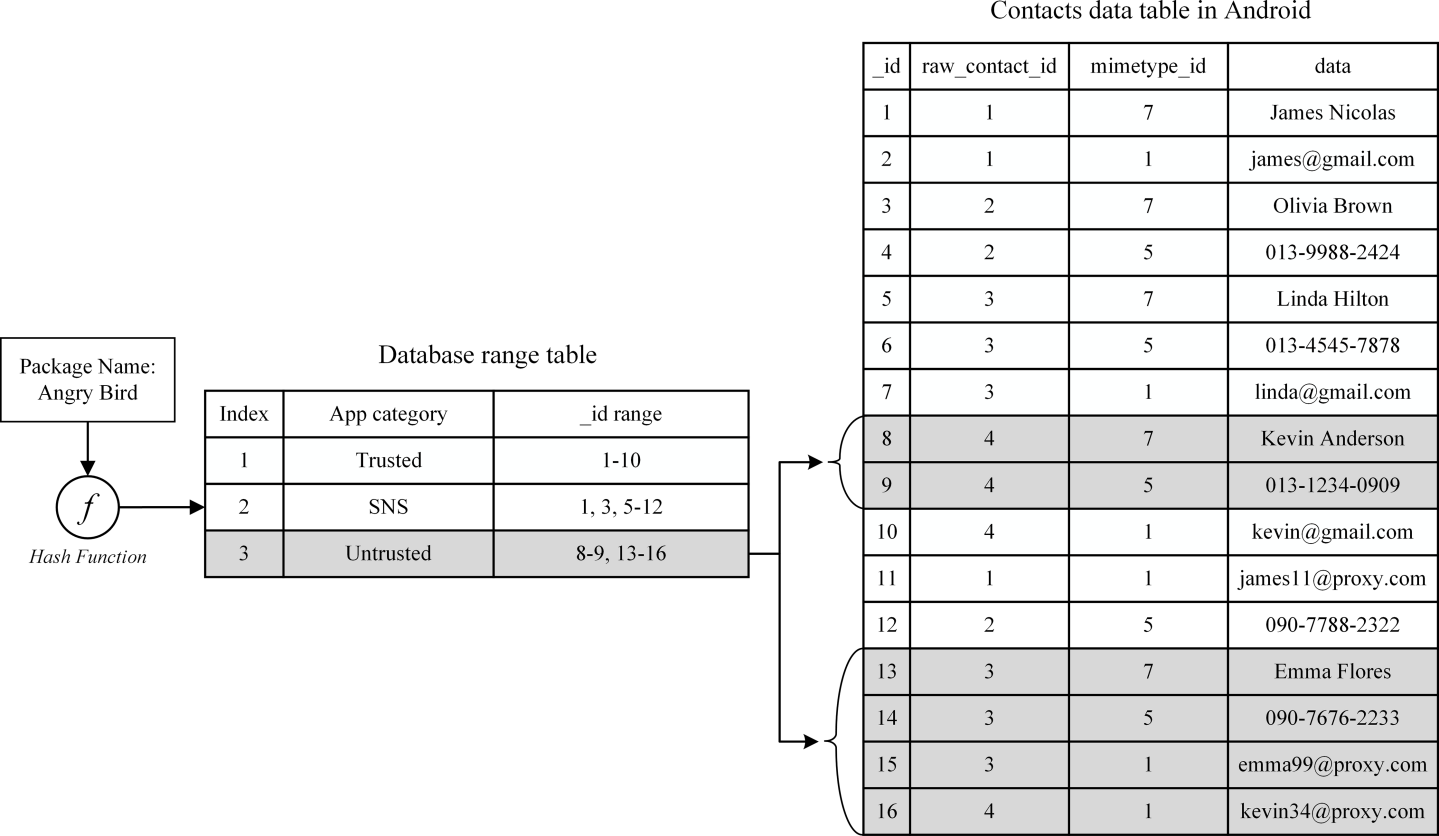
An example of finding original and virtual data accessible from Angry Bird belonging to an untrusted app in the proposed contacts’ database using the database range table.

**Fig 8:**
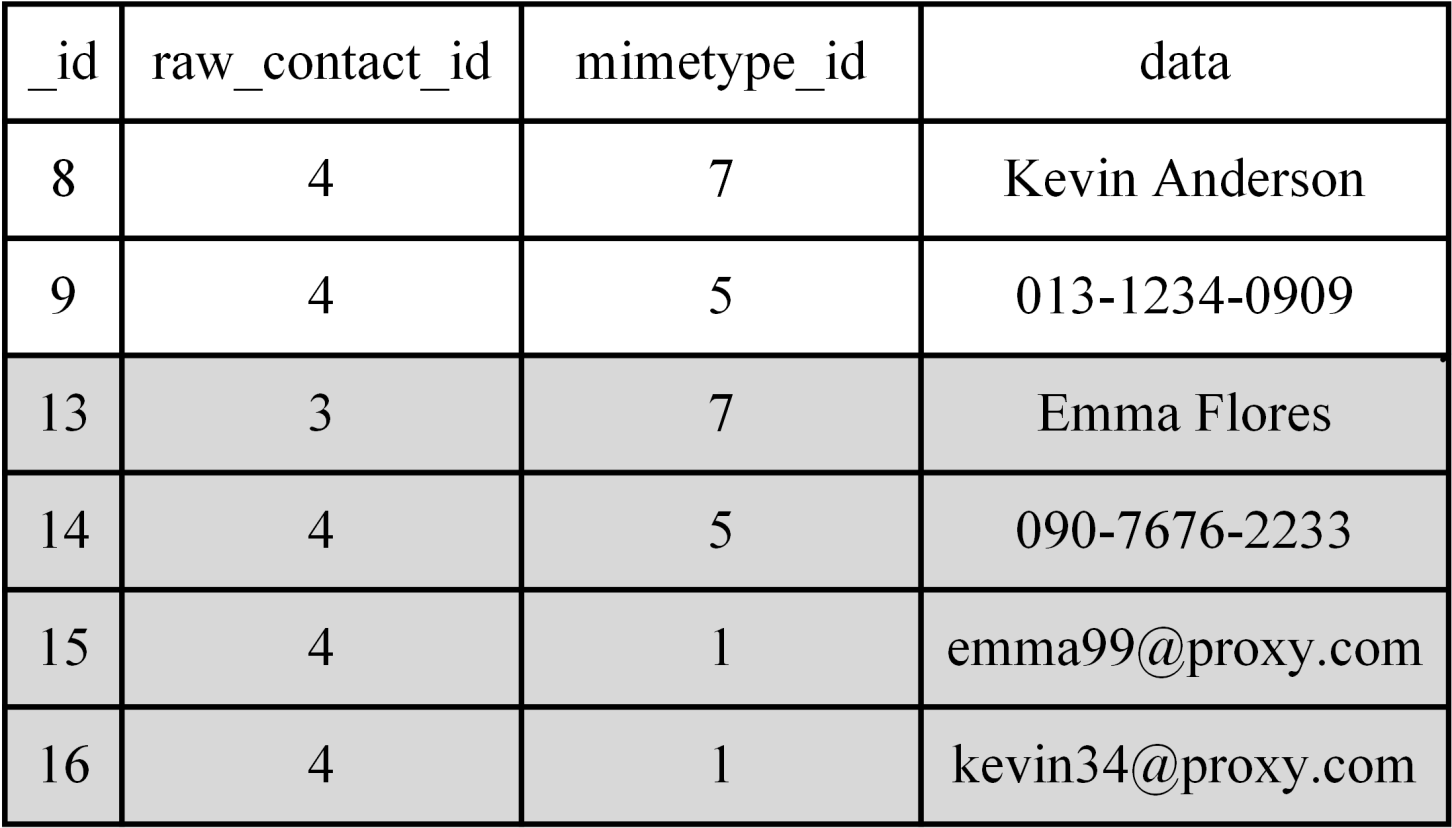
Contacts from the untrusted apps’ category.

The virtual data are located below the original data. Such a consecutive arrangement of data allows the system to retrieve all related database records for a specific app category without increasing access time. For example, we use the following SQL query to create an untrusted view for Angry Bird as shown in Fig 6:

To build a new SQL query for each app category, we need to find the ranges of indices of the original and virtual data. We can easily do this by using a hash-based data structure called the ‘database range table,’ as shown in Fig 7. We can hence build each ‘view’ while launching the corresponding app without a significant delay that can bother users. Fig 8 shows the final view for an untrusted app category for the SQL query in Fig 6 for the contacts’ data shown in Fig 7.

### Private data management server

The private data management server is responsible for allocating and managing private data when a user configures the proposed system using the policy configuration app. Virtual data are used to detect and track leaked private data; thus, the virtual data should be uniquely generated for each <contacts category, app category> pair.

When data is leaked, the server notifies the e-mail proxy and the virtual phone number servers of this, and the leaked virtual data are discarded to prevent subsequent damage, such as e-mail spam or voice phishing.

### E-mail proxy server

The e-mail proxy server relays e-mails destined to virtual addresses created by the private data management server to their real destinations. It internally maintains a database consisting of virtual and real e-mail addresses.

The proposed system uses ‘Sendmail’ and ‘Milter’ to build the e-mail proxy server [78, 79]. Milter acts as a filter that inspects received e-mails and processes them according to policies transmitted by the private data management database.

The user can configure the e-mail proxy server to insert a warning message into the subject of the e-mail and relay it to the real destination, or to transmit a leakage detection message without any content in the body of the e-mail to the destination. The message contains the name of the app category to which the leaking app belongs, thus enabling the user to easily detect and handle malicious apps. Depending on the policy, it may also leave a log message and discard the e-mail.

This approach causes a delay of less than a few seconds; but e-mail is not real-time service and, therefore, such delay should not affect user experience.

### Virtual phone number server

Similarly to the safe number service, a virtual phone number server relays calls or short message service (SMS) messages destined for a virtual phone number to real one. It differs from existing safe number services in that it can deliver a warning message prior to call setup and insert similar texts into SMS messages.

It can also disconnect logical connections between virtual and real phone numbers, and can send a leakage detection message to the user, as with the e-mail proxy server.

Call relay through a virtual phone number server creates additional delay, and it is critical to ensure that this delay is short to prevent users from becoming agitated. We can estimate the delay based on existing safe number services. In experiments, we found that the delay was less than a second. Thus, this approach can effectively protect users from private leaks without additional inconvenience.

### How the system works

To describe the overall operation of the proposed system, we assume that a user uses a smartphone through the system shown in Fig 8. E-mail addresses and phone numbers are handled by the e-mail proxy and the virtual phone number servers, respectively, but the two processes are similar. Therefore, we only describe the detailed procedure for virtual e-mails, where the reader should assume that the same description represents, mutatis mutandis, the procedure for virtual phone number servers.

User A configures a policy and an external private data management server generates virtual data for fields such as name, e-mail address, or phone number. The generated data are accordingly transmitted to the external e-mail proxy or the virtual phone number servers.

Suppose a malicious app leaks and transmits contacts’ data from user A’s smartphone to an external spam server. We also assume that the leaked data for a user B, among the contacts of user A, are virtual. Thus, the virtual e-mail address of user B has been leaked.

**Fig 9:**
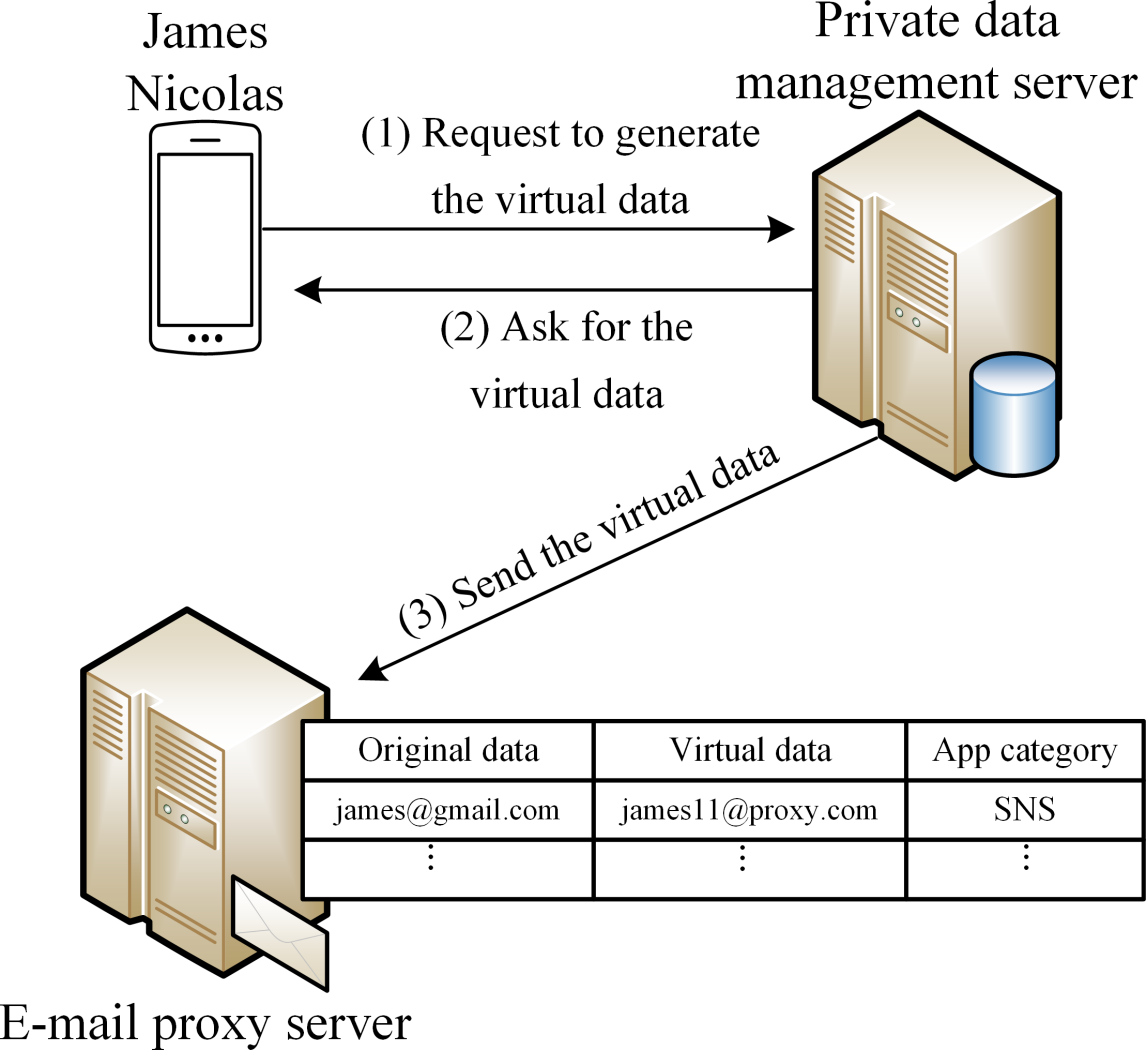
Creation flow of virtual private data for e-mail.

**Fig 10:**
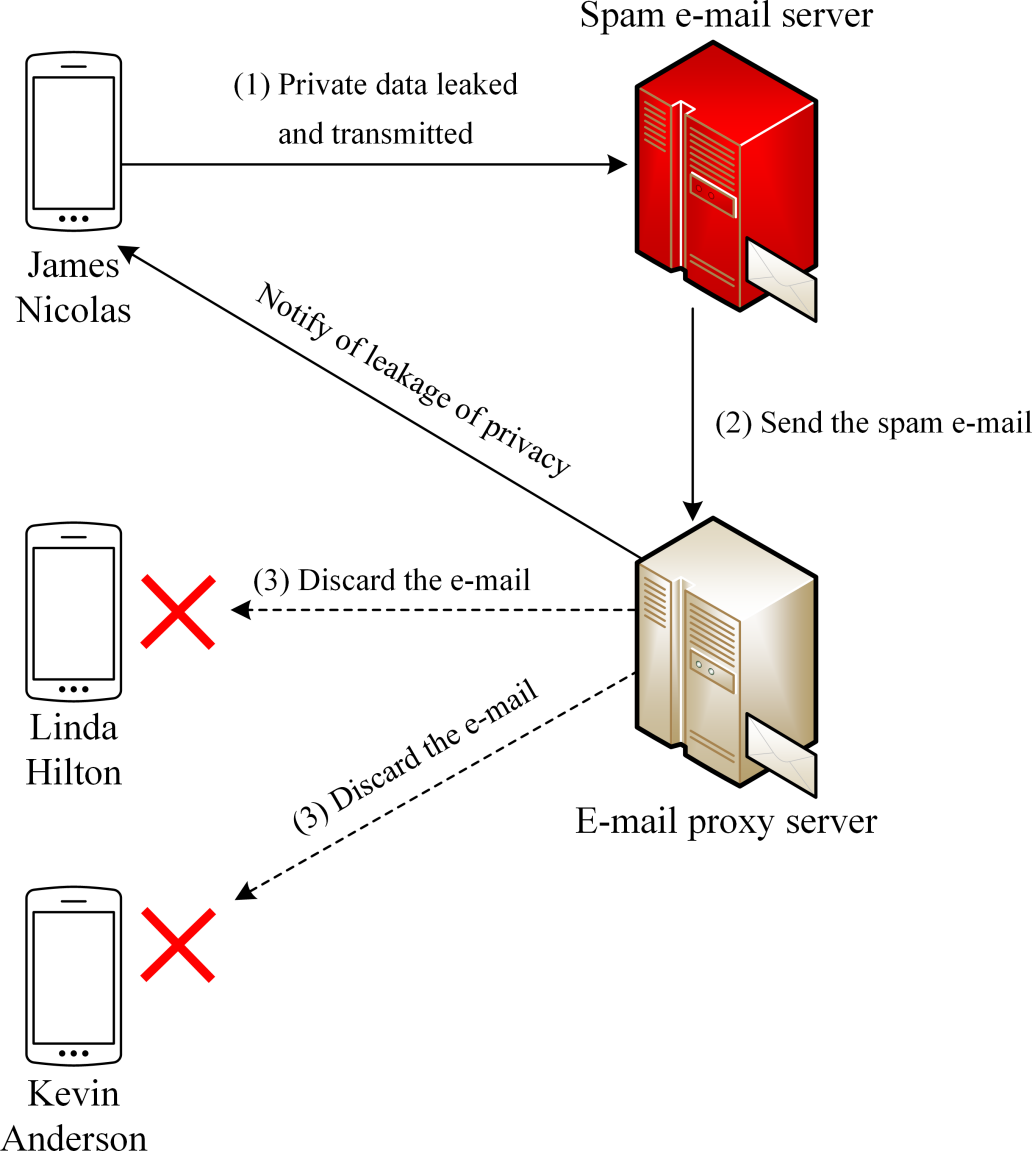
Detection of private data leakage.

The leaked e-mail address of user B is used for spam as shown in Fig 9. Since the address is the virtual address of the e-mail proxy server, spam e-mail is destined for the e-mail proxy server instead of user B. If the policy for data pertaining to user B is ‘discard and notify,’ the proxy server discards the spam e-mail and sends a message to user A to the effect that the contacts’ data of user A has been leaked, as shown in Fig 10.

The message also contains the name of the app category to which the malicious app belongs and hence helps the user determine what the app is. Moreover, user B does not receive any spam e-mail even though his/her information was leaked, and is hence protected.

## Performance evaluation

We conducted experiments on the Samsung Galaxy S5 smartphone for accurate performance evaluation and analysis of the proposed system. We implemented the system using CyanogenMod Android Lollipop 5.0.2 source code. Since the proposed approach is different from prevalent solutions, it was difficult to choose competitors. Our system provides very similar security to that offered by KNOX if the external e-mail proxy and the virtual phone number servers are excluded. Therefore, we chose KNOX for comparison. We also chose MockDroid since it has similarity in that it uses policy to control accessing private data [62].

We used three scenarios for the assessment. For each, the number of contacts was increased from 50 to 200 to analyze scalability. We configured two categories of contacts—‘Family’ and ‘Friend.’ The ratio of the number of contacts in ‘Family’ to that in ‘Friend’ was 3:7. For example, if the total number of contacts is 100, 30 belong to ‘Family’ and 70 to ‘Friend.’ The number of app categories was increased from two to 10, and policies were differently configured for them according to scenario. To make the performance evaluation easy, we built a script file that can be found in S1 Zip. The script automatically runs each evaluation one by one.

(1) Scenario 1

‘Hiding’ and ‘Virtualization’ were set for all fields of contacts, such as name, e-mail address, and phone number, belonging to ‘Family’ and ‘Friend,’ respectively.

(2) Scenario 2

‘Virtualization’ was set for all fields of contacts belonging to ‘Family’ but only for the e-mail addresses of contacts in ‘Friend’.

(3) Scenario 3

‘Virtualization’ was set for the e-mail addresses of contacts in ‘Family’ but to ‘No-protection’ for all fields of contacts in ‘Friend’.

### Size of the contacts’ database

#### Size of contacts’ database according to the size of contacts

We compared the size of the contacts’ database of the proposed system when one app category was used in Scenario 2 with those of KNOX and MockDroid when one container was used.

In the experiment, each system, logically or physically, had two contacts, i.e., one in Android and the other in the container for KNOX, or one for the first app category and the other for the second app category. Thus, it was fair to compare the total size of contacts. For better situation for KNOX, only contacts in the second app category were saved as contacts in KNOX.

**Fig 11:**
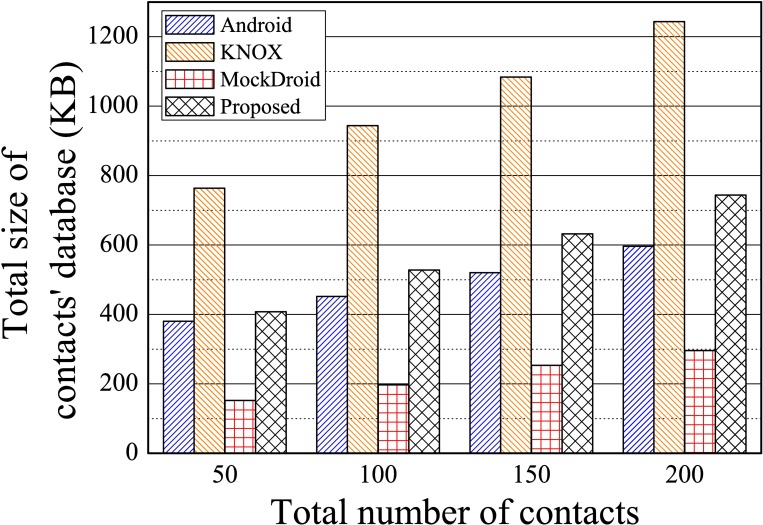
The size of the contacts’ database according to the number of contacts when one app category and one container were used for Scenario 2.

The results of the experiment are shown in Fig 11. The size of the contacts’ database for Android without KNOX is also shown for analysis.

As shown in Fig 11, the database of the proposed system was at least 60% smaller in size than that of KNOX and only 25% larger than that of the Android system. This confirmed that our system can provide security without significant storage overhead. Since most smartphones have limited local storage, the proposed system offers an efficient solution.

#### Size of contacts’ database according to number of app categories

The size of the contacts’ database increased proportionally to the number of app categories. However, the more app categories a user has, the more accurately the proposed system can detect a malicious app leaking private data. Therefore, even if the number of app categories increases, it is important to prevent the size of the database from increasing significantly.

We measured the size of the contacts’ database of the proposed system according to the number of app categories, with 200 contacts in the database. Since KNOX and MockDroid cannot support multiple containers or categories, they were excluded from this evaluation.

**Fig 12:**
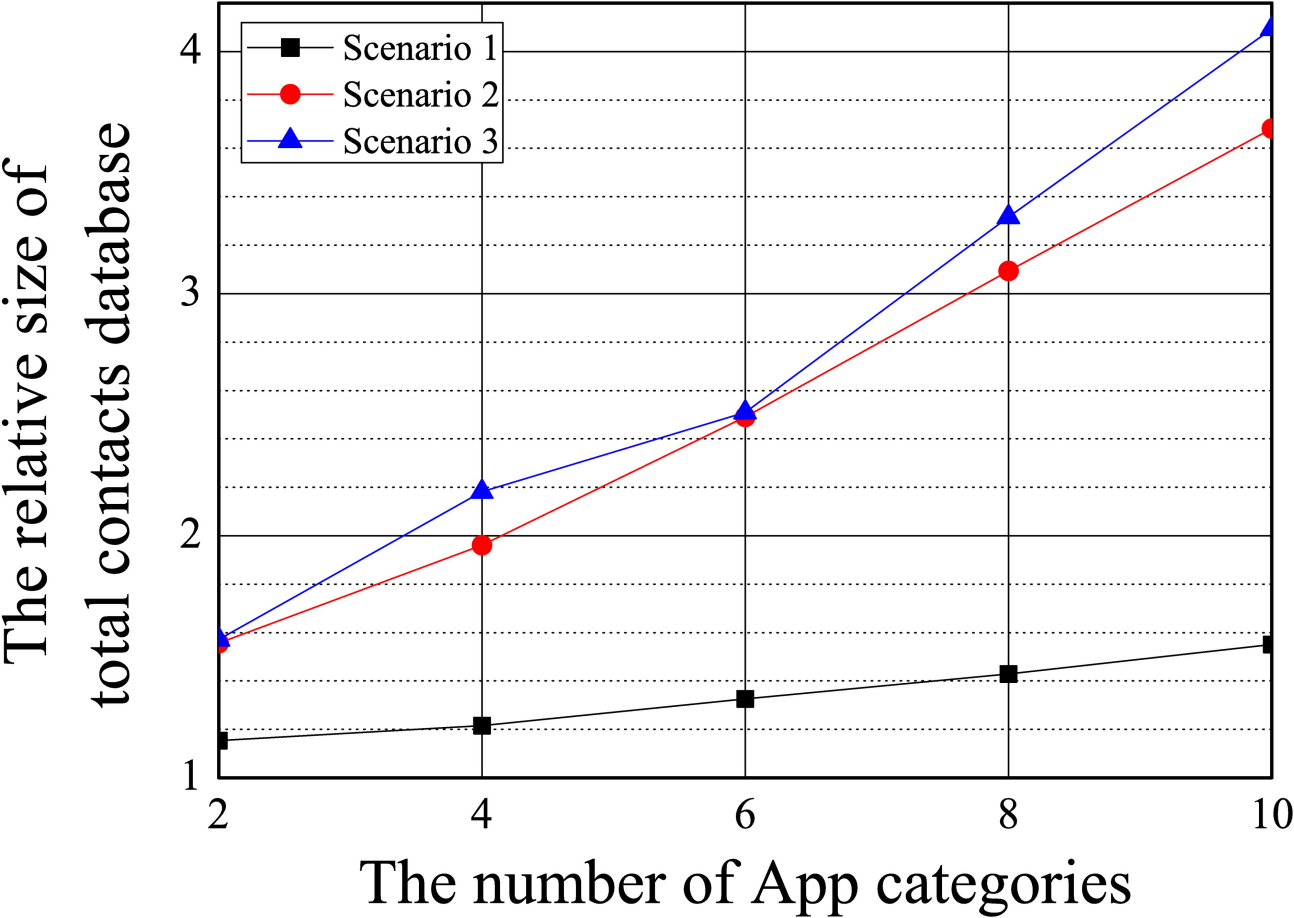
The relative size of the contacts’ database compared with that in the original Android, where the number of contacts was 200.

Fig 12 shows the relative measured size of the total contacts’ database for different numbers of app categories by assuming that the size of the contacts’ database with 200 contacts was one.

In Fig 12, we see that the increase in the size of the database varied according to scenario because of the different numbers of virtual data items in each. For example, contacts in the ‘Friends’ category were not protected in Scenario 3, so no virtual data need for the category.

For cases involving 10 categories, the size of the database in the worst case was only 4.1 times larger than that of the Android database. This showed that the proposed system can support a large number of app categories without significant cost in terms of storage space. As mentioned above, more app categories increase the accuracy of the detection of malicious apps. Hence, it is a critical advantage of our system that it can support a large number of app categories.

The proposed system minimizes the increase in storage overhead while supporting multiple app categories using the ‘view’ instead of the ‘table’. Table 1 shows the total storage for ‘table’ and ‘view’ according to the number of app categories when the number of contacts was 200. Since each contact had three records—name, e-mail address, and phone number—the total number of records was 600.

**Table 1:** Database size according to the number of categories of apps for Scenario 2.

Number of App categories	Database Size
0	610,304
1	618,496
2	622,592
3	626,688
4	630,784
5	634,880
6	638,976
7	643,072
8	647,168
9	651,264
10	655,360

Table 1 shows that database size increased by only 4 KB, except in the first case, as the number of app categories increased by one. Since this was only 0.6% of the size of the entire database, this shows that the ‘view’ is much more efficient in terms of storage than ‘table’.

**Fig 13:**
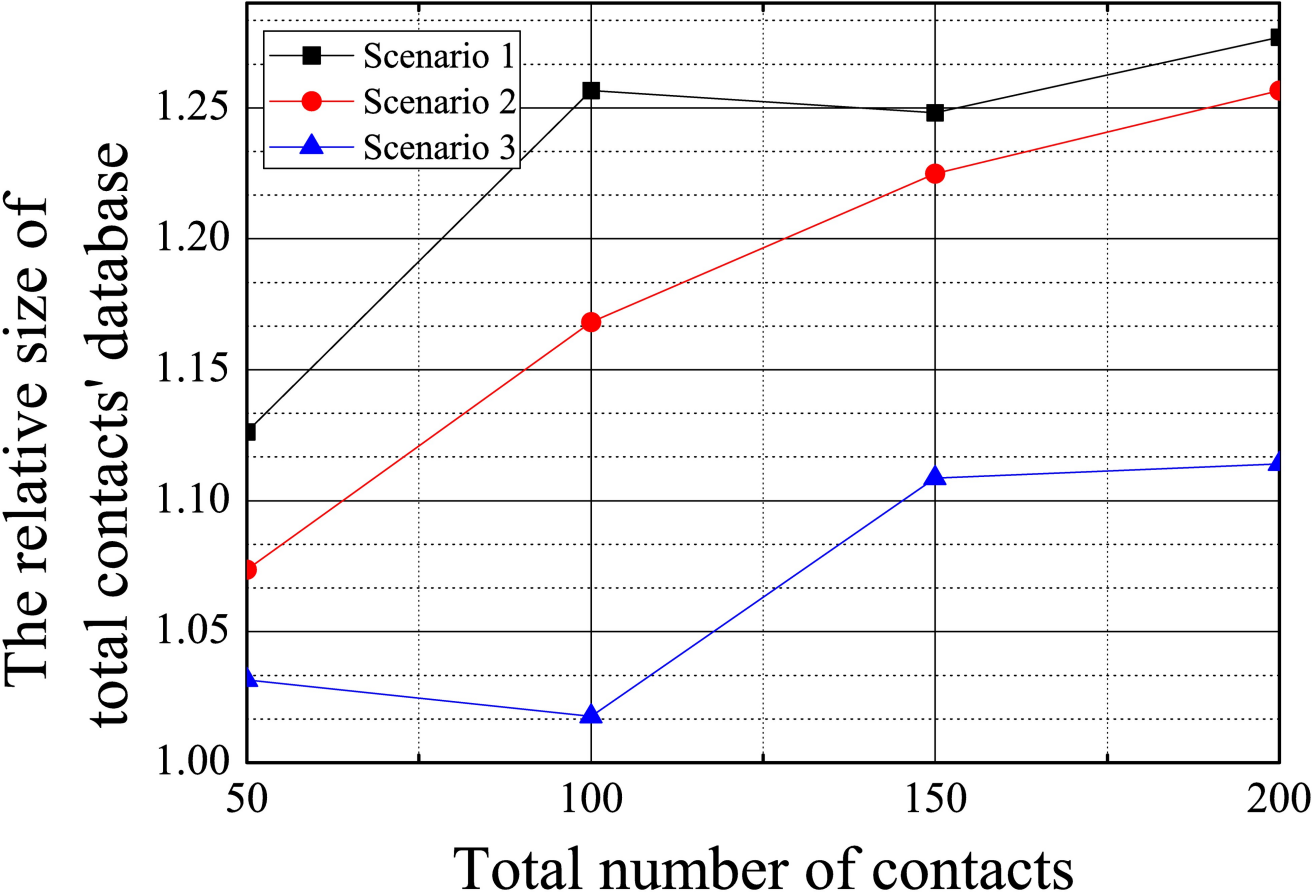
The ratio of the size of the contacts’ database of the proposed system to that of the Android system according to the total number of contacts.

Fig 13 shows the increase in the size of the contacts’ database of the proposed system compared to the Android as the total number of contacts increased. For all scenarios, the ratio of increase was lower than 1.3; thus, the proposed system efficiently uses the database table.

### Performance in terms of accessing contacts’ database

**Fig 14:**
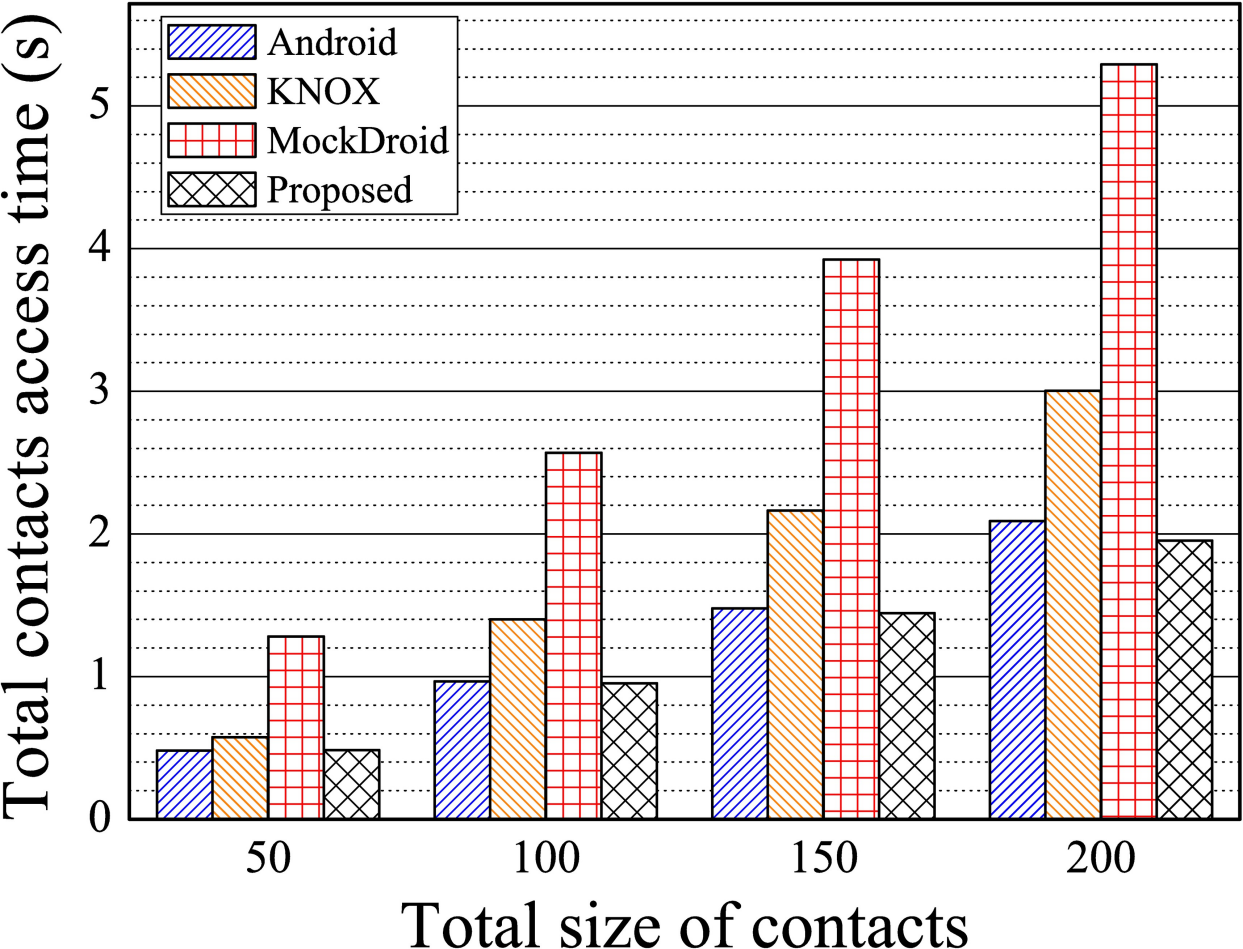
The total time of access to contacts according to the number of contacts, where one app category and one container were used for Scenario 2.

To compare the access performance of the proposed system with that of KNOX, we measured the total access time needed to read every data item in the contacts’ database according to number of contacts. As shown in Fig 14, the proposed system delivered almost the same performance as the Android, but the results for KNOX were 50% poorer due to overhead from the container. MockDroid showed the same performance as the original Android since it functions identically to the Android when a mocked permission is not applied. The proposed system used a prebuilt view for each app category, thus guaranteeing comparable performance to the Android.

**Fig 15:**
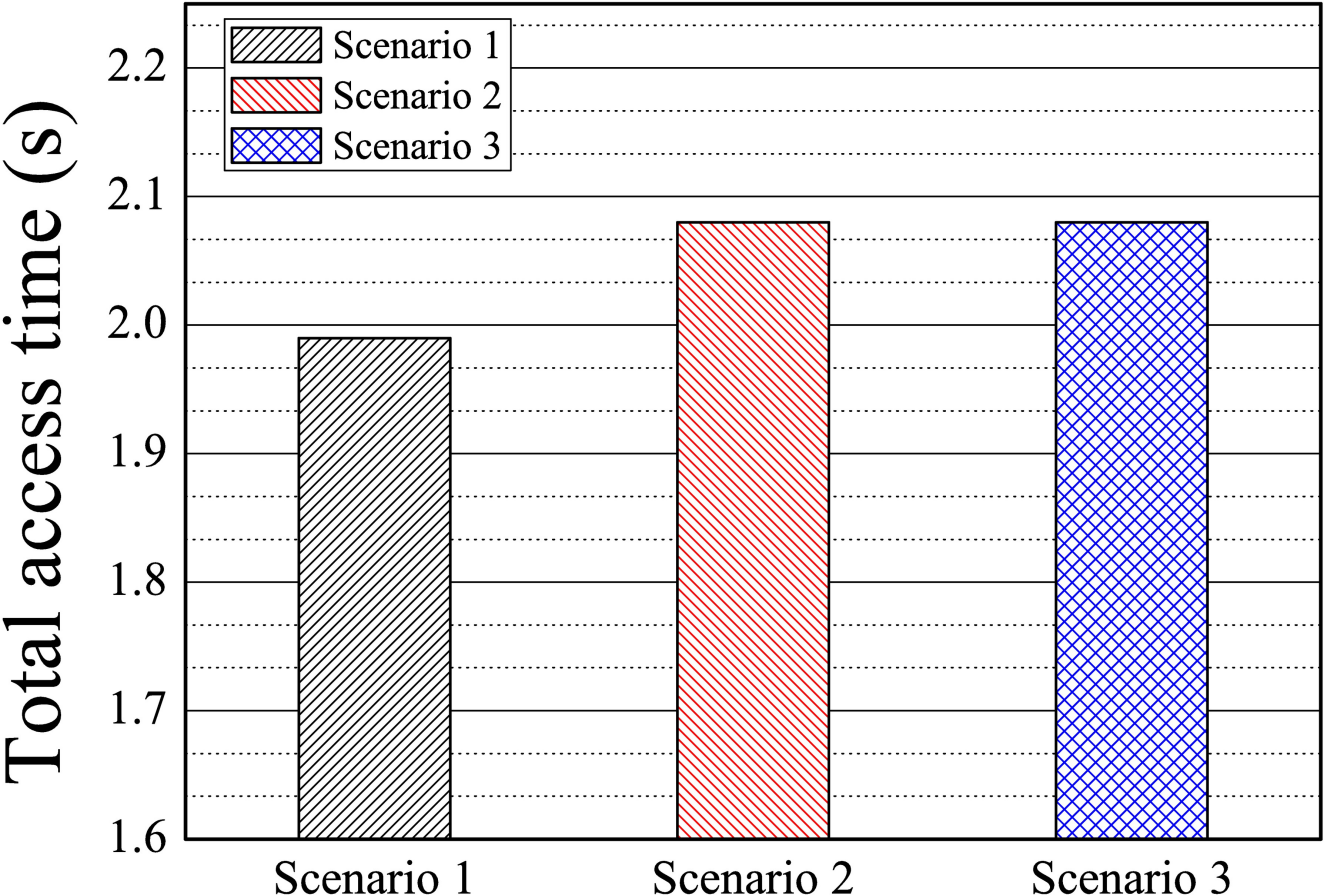
The total time of access to 200 contacts.

Fig 15 shows the total access time taken to read 200 contacts for each scenario. Scenario 1 had the fastest access time as the policy ‘hiding’ was applied to the contacts’ category ‘Family,’ so that the number of contacts in the view was the smallest of all scenarios. It took only 10ms to read each contact on average regardless of scenario, thus confirming that our system guarantees short access time.

## Conclusions

The proposed system has the unique feature whereby it can apply various security policies to different app categories. It can achieve almost the same access performance as the Android system and has a smaller database compared to competitors.

If attackers try to directly access a database file, they can bypass the security mechanism of the proposed system. However, many studies have proposed mechanisms to protect file systems, and hence the problem can be simply solved by combining such solutions with the proposed system.

Our system uses an external e-mail proxy and virtual phone number servers to prevent private data from leaking and detect malicious apps. Other platforms besides Android can use the system without modification. Therefore, we expect it to play an important role in protecting private data in various environments.

## Supporting information

S1 Zip The script file to build the proposed Android from CyanogenMod Android Lollipop 5.0.2 source code, and automatically conducts experiments. (ZIP)
